# A Comparative Immunohistochemical Study of Wound Healing after Dental Diode Laser Treatment in the Rat Oral Mucosa

**DOI:** 10.3390/bioengineering9090466

**Published:** 2022-09-13

**Authors:** Hye Rin Kim, Keunbada Son, Young-Tak Son, Yong-Gun Kim, Kyu-Bok Lee, Seung Cheol Lee, Jo-Young Suh, Jae Mok Lee

**Affiliations:** 1 Department of Periodontology, School of Dentistry, Kyungpook National University, Daegu 41940, Korea; 2 Advanced Dental Device Development Institute (A3DI), Kyungpook National University, Daegu 41940, Korea; 3 Department of Dental Science, Graduate School, Kyungpook National University, Daegu 41940, Korea; 4 Department of Prosthodontics, School of Dentistry, Kyungpook National University, Daegu 41940, Korea; 5 Department of Pathology, Andong General Hospital, Andong-si 36743, Korea

**Keywords:** dental diode laser, histologic research, periodontal surgery, scalpel, wound healing

## Abstract

This study aimed to examine the differences in healing patterns using two types of diode laser devices (laser A and laser B) and a steel scalpel for periodontal surgery through histological and immunohistochemical methods. Twenty 12-week-old male rats were assigned to three groups (3, 7, and 14 days). Square-shaped erosion wounds (2 × 2 mm^2^ diameter) were created on the hard palate of each rat. Two wounds were created using Laser A and a steel scalpel (Bard-Parker No. 15) on the right palate and using Laser B and a steel scalpel on the left side. Rats were sacrificed after 3, 7, and 14 days. Tissues were collected with a margin of 1 mm from the border of the erosional wound of the maxillary hard palate. Histological and immunohistochemical analyses were performed on the tissue samples after 3, 7, and 14 days. The tissue healing pattern and expression of inducible nitric oxide synthase (iNOS) and cluster of differentiation (CD) were observed under a light microscope. Statistical analysis was conducted using the Kruskal–Wallis H test for comparison among the groups (α = 0.05). In comparison to the wounds made with the scalpel, wounds treated with lasers A and B showed delayed healing patterns. There was no significant difference between the two laser treatment groups (*p* > 0.05). The expression of iNOS and CD68 was not significantly different among the three groups after 3 and 7 days (*p* > 0.05). On day 14, the groups treated with the dental diode lasers showed higher expression than the group treated with the steel scalpel, but no significant difference was observed (*p* > 0.05). Laser-induced wounds tended to heal slower than surgical wounds performed using a steel scalpel, but histological and immunohistochemical results showed no significant difference between the dental diode laser and scalpel groups.

## 1. Introduction

The steel scalpel is the most widely used instrument in soft tissue surgery because of its ease of use, accuracy, and minimal damage to adjacent tissues. However, scalpels have critical disadvantages in that they do not provide hemostasis in places where a large number of blood vessels are distributed, such as in the oral cavity [1]. To overcome this problem, electrosurgery and lasers have been introduced and are actively used in oral surgery. Lasers were first used in the dental field in the 1960s. Initially, lasers were used only for the treatment of hard tissue and for caries removal. However, it has recently been used for soft tissue treatment [2]. In soft tissue surgery, lasers offer the advantages of local hemostasis, bacterial elimination, and contact-free incision [3]. Postoperative pain is also significantly reduced with the use of lasers. However, excessive exposure to laser energy can cause tissue damage and destruction, highlighting the importance of establishing a proper laser application method.

Lasers used in dental treatment include CO_2_, Nd:YAG, Er:YAG, ER,Cr:YSGG, dental diode lasers, and argon lasers. Dental diode lasers have a wavelength of 655–980 nm and are used for periodontal surgery such as gingivectomy, gingivoplasty, and implant second surgery.

Wound healing is the basic mechanism for restoring tissue integrity after injury. The process of wound healing after periodontal surgery can be divided into primary and secondary healing. The healing that occurs when the margin of the wound is rejoined with minimum space through the closure of the valve is called primary healing. Wounds fused during primary healing showed minimal tissue loss. However, when the flaps are not completely joined or the de-epithelialization procedure for pigment removal is performed, the wound is restored to a state where the margins are not adjacent, that is, secondary healing occurs [4]. As a significant amount of fibrosis occurs during the process of filling the tissue defect with new connective tissue, scar formation due to contraction is more pronounced than in the case of primary healing [5].

Wound healing is a dynamic process that involves a variety of cellular and biological mediators. The wound healing process involves three stages, namely, the inflammatory stage, granulation tissue formation stage, and maturation stage, each of which is orchestrated by various types of inflammatory cells, cytokines, and biologically active molecules such as tissue growth factors. The onset of traumatic injury results in immediate capillary damage and excessive bleeding, leading to the formation of blood clots. Several growth factors that appear in blood clots regulate the generation of granulation tissue by mobilizing inflammatory cells. Inflammatory cells, including neutrophils and monocytes, migrate to the blood clot within a few hours after injury and remove bacteria and necrotic tissues from the wound through phagocytosis and secretion of toxic oxygen products [6]. At a later stage of the inflammatory response, macrophages migrate to the wound site and secrete inflammatory cytokines, allowing more inflammatory cells to migrate to the wound site. Cluster of differentiation 68 (CD68) is a cell-penetrating glycoprotein expressed at elevated levels in monocytes (e.g., monocytic phagocytes, osteoclasts) and macrophages [7,8]. CD68 is used as a marker of monocyte/macrophage-related inflammatory responses in histological studies [9].

Nitric oxide (NO) is an important biological signaling molecule involved in the regulation of platelet function, blood flow, and macrophage cytotoxicity. It also affects innate immunity [10,11]. NO is produced by NO synthase (NOS). Three isoforms of NOS have been identified, including endothelial NOS (eNOS), neuronal NOS (nNOS), and inducible NOS (iNOS). iNOS plays an important role in the inflammatory response, and unlike eNOS and nNOS, it continuously produces a large amount of NO [12].

The purpose of this study was to investigate the histological and immunohistochemical differences in the rat oral mucosa after de-epithelization using a steel scalpel and two dental diode lasers. Healing was evaluated after 3, 7, and 14 days using light microscopy and immunohistochemically by measuring iNOS and CD68 levels. The null hypothesis of the present study was that there is no difference in the immunohistochemical results for wound healing after treatment with the steel scalpel and two dental diode lasers.

## 2. Materials and Methods

### 2.1. Dental Diode Laser Devices

**Figure 1:**
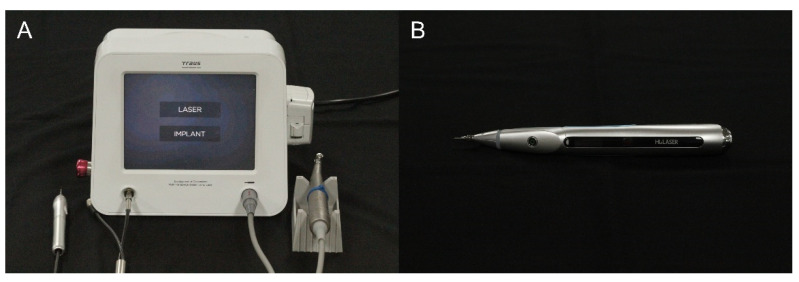
Dental diode laser device used in the present study. (**A**) Laser A group. (**B**) Laser B group.

In this study, two dental diode lasers were employed in a continuous mode, emitting light at a wavelength of 980 nm. Laser A (dental diode laser; Saeshin, Daegu, Korea) was of a type connected to the laser/implant integrated module by a wire; a fiberoptic tube was attached to the end of a handpiece with a tip 400 μm in diameter and 45 mm in length (Figure 1). Laser B (K2 mobile; HULASER, Seoul, Korea) was also a continuous mode/pulse mode diode (GaAlAs) laser with an optic fiber tip 400 μm in diameter (Figure 1). Laser radiation was applied to the oral mucosa of rats at 3 W output power and 30 J of total irradiation energy. The tip was applied in direct contact with the epithelial surface during the laser exposure.

### 2.2. Animal Model and Study Design

**Figure 2:**
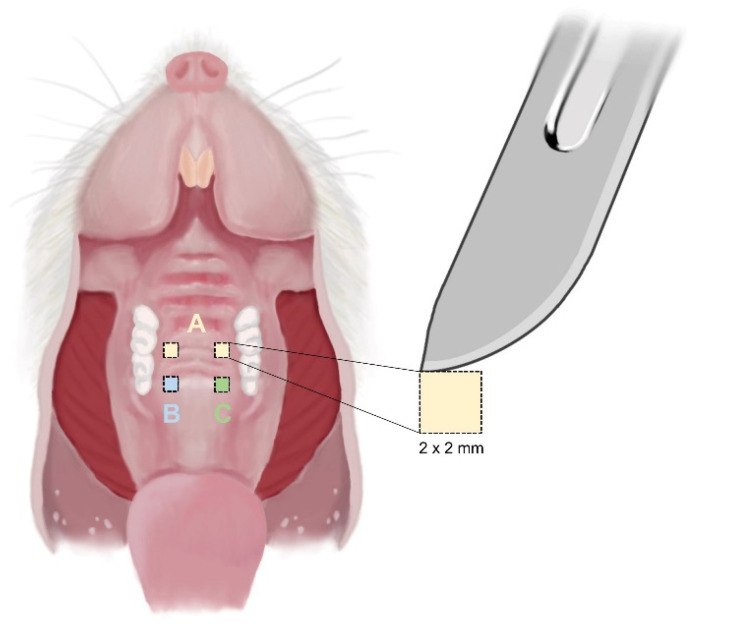
Schematic illustration showing erosion wound in the oral cavity in the rat. (A) Steel scalpel. (B) Laser A. (C) Laser B.

This experimental study was approved by the Animal Ethics Committee (IACUC) of the Daegu Kyungpook Advanced Medical Industry Promotion Foundation based on the Animal Protection Act (Act No. 4379 of 31 May 1991, partially revised Act No. 12053 of 20 January 2015) (Approval No.: DGMIF-00). Fifteen 12-week-old male rats were housed in metal cages (two each) and maintained at room temperature for 12 h per day with light and 50% relative humidity. The rats were provided with an ad libitum supply of solid food and water through a feeding machine. Before the experimental procedure, rats were randomly divided into four groups. Six of the 15 rats were assigned to preliminary experiments, and nine rats were assigned to three groups (3 rats in each group; 3, 7, and 14 days). Then, 2 mm by 2 mm square-shaped erosion wounds were created on the hard palate of each rat. Two wounds were created using Laser A and a steel scalpel (Bard-Parker No. 15) on the right palate and using Laser B and the steel scalpel on the left side (Figure 2). The three wounds were sutured or treated with dressings. After surgery, the mice were sacrificed at intervals of 3, 7, and 14 days, and tissues were analyzed to compare histological and immunohistochemical changes in each group at different time points.

### 2.3. Presurgical Treatment and Surgical Procedure

Rats were anesthetized by injection of Zoletil 50 solution (chloral hydrate tiletamine chloral hydrate zolazepam 50 mg/mL; Verbac Laboratories, Carros, France) and Rompun (23.32 mg/mL) at a dose of 80 mg/kg body weight. After anesthesia, the surgical site in the rat oral cavity was sterilized with betadine solution (Viatris GmbH & Co. KG, Frankfurt, Germany). Two types of wounds were created using a steel scalpel and two dental diode lasers (Laser A and B). As mentioned above, the rats were divided into three groups (3, 7, and 14 days). In the oral cavity of all subjects, two types of erosion wounds were made by using a scalpel and Laser A at the right hard palate. On the other hand, two wound sites were formed on the left hard palate using scalpel and Laser B, respectively. Sutures were not performed.

Two diode lasers were used with continuous output power of 3 W and wavelength 980 nm. The energy density was 750 J/cm^2^ (total energy 30 J). The laser tips of two devices were initiated by touching the tip to the articulating paper. During irradiation, the laser tip beam was in contact with the irradiated tissue surface and continuously moved to prevent overheating, necrosis, and carbonization of the tissue (for 10 s). During the application of surgical instruments, the blood was frequently suctioned using a syringe. All the surgical procedures were performed by the same operator under aseptic conditions. After surgery, the rats were placed on a heating pad until they recovered from anesthesia.

In case of active bleeding after wound formation, hemostasis was performed by compressing the wound with gauze or using hydrogen peroxide. Then, rats were returned to the cage.

### 2.4. Postsurgical Treatment and Histological Procedures

To prevent postoperative infection and reduce pain, gentamicin and metacam were administered for 4 days after surgery. The rats were sacrificed on days 3, 7, and 14 after surgery. After taking pictures, the wound site was visually observed, and the healing process was evaluated. Specimens with margins of up to 1 mm from the outside of the wound were collected from both sides of each rat. The samples were immediately fixed in 10% neutral-buffered formalin. After embedding in paraffin wax, a section was prepared across the center of each wound and stained with hematoxylin and eosin (H&E).

The tissue healing pattern was observed under a light microscope, and the presence of cells related to the inflammatory response was investigated.

### 2.5. iNOS and CD68 Immunohistochemical Procedures

To observe the expression of iNOS and CD68, immunohistochemical staining was performed on sections adjacent to the H&E-stained sections. The sections were treated with a blocking agent containing 5% normal goat serum and 0.1% serum albumin in phosphate-buffered saline (PBS) at room temperature for 30 min. Thereafter, the sections were rinsed in TRIS-HCl for 5 min and incubated with primary rabbit antibodies for iNOS and CD68 (Santa Cruz Biotech Inc., Santa Cruz, CA, USA) for 15 min. After incubation with the primary antibody, the sections were rinsed with TRIS-HCl and incubated with a secondary goat anti-rabbit antibody for 30 min. The sections were then rinsed and incubated with the peroxidase-antiperoxidase complex for 10 min. Antibody complexes were visualized as red precipitates.

### 2.6. Evaluation of Wound Healing and Histomorphometry

After H&E and immunohistochemical staining, the specimens were analyzed using optical microscopy (BX43; Olympus Corporation, Tokyo, Japan). Ulceration severity was classified as (absent) or (present). The infiltrated neutrophils, histiocytes, and lymphocytes were counted under a microscope. Cells that were positive for iNOS and CD68 expression were also counted. The number of cells in the field viewed at 100× magnification was classified as 0 (0 cells/HPF), 1 (1–30 cells/HPF), 2 (31–60 cells/HPF), and 3 (more than 61 cells/HPF).

### 2.7. Statistical Analysis

IBM SPSS Statistics for Windows, version 25 (IBM Corp., Armonk, NY, USA) was used to analyze all data (α = 0.05). First, the distribution of the data was investigated using the Shapiro–Wilk test; the data were not normally distributed. Statistical analysis was conducted using the Kruskal–Wallis H test for comparison among treatment groups (scalpel, laser A, and laser B groups) or days (3, 7, and 14 days).

## 3. Results

### 3.1. Visual Assessment

**Figure 3:**
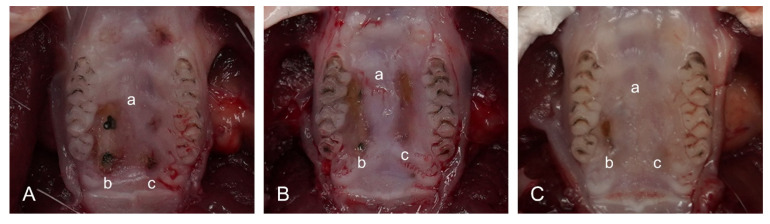
Photos taken at 3 days (**A**), 7 days (**B**), and 14 days (**C**) after surgery; a: steel scalpel, b: laser A group, c: laser B group.

There was no significant difference in the degree of bleeding during wound formation between groups. In the area where the erosion of the maxilla was formed, the region treated with Laser A and Laser B showed a slower healing pattern than that treated with the steel scalpel. In addition, some wounds did not completely heal by day 14 in the laser groups (Figure 3). There was no significant difference in the healing patterns between the two dental diode lasers.

### 3.2. H&E Staining

**Figure 4:**
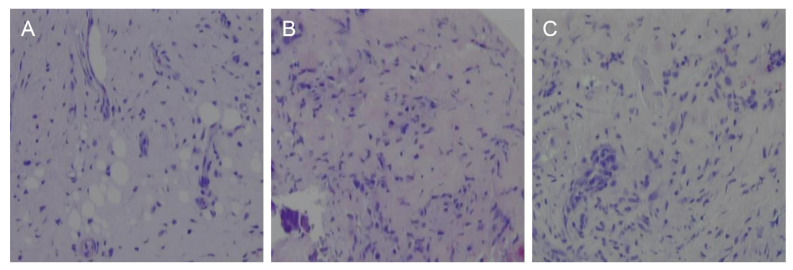
The inflammatory cell infiltration was higher in the H&E-stained samples of the rat palate from the laser A (**B**) and laser B (**C**) groups than those from the rats exposed to the scalpel wound (**A**) 3 days after surgery.

In the H&E-stained samples, the presence or absence of ulcers and the number of neutrophils and lymphocytes in the specimens were counted at 100× magnification (Table 1). In the group exposed to the steel scalpel, ulceration was not observed in any of the specimens. On day 3, ulcers were observed in both the laser A and laser B groups, but the frequency was higher in the laser A group than in the laser B group. The presence of neutrophils and lymphocytes was not significantly different between the three groups at any interval (Figure 4).

### 3.3. Immunohistochemical Staining

**Figure 5:**
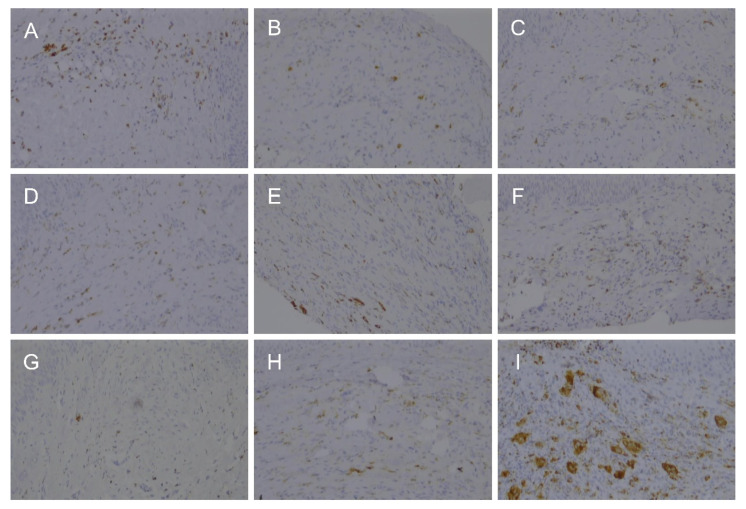
Expression of CD68 in the rat palate tissue after 3 days (**A**–**C**), 7 days (**D**–**F**), and 14 days (**G**–**I**). Scalpel (**A**,**D**,**G**), laser A (**B**,**E**,**H**), and laser B (**C**,**F**,**I**).

The presence of histocytes was investigated using immunostaining for CD68. The number of histocytes did not differ between the three groups on days 3 and 7, but was higher in the two laser-treated groups on day 14 (Table 1 and Figure 5).

**Figure 6:**
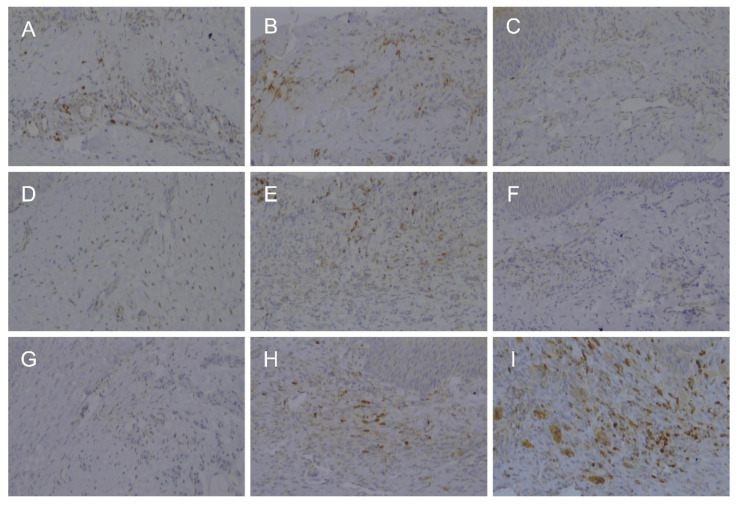
Expression of iNOS in the rat palate tissue 3 days after 3 days (**A**–**C**), 7 days (**D**–**F**), and 14 days (**G**–**I**). Scalpel (**A**,**D**,**G**), laser A (**B**,**E**,**H**), and laser B (**C**,**F**,**I**).

Similarly, the number of iNOS-positive cells was not different among any of the groups on days 3 and 7. However, after 14 days, the expression of iNOS tended to decrease in the group treated with the steel scalpel, whereas the two groups treated with the laser still showed high iNOS expression (Table 1 and Figure 6).

## 4. Discussion

The purpose of the present study was to treat surgical wounds in rats using a steel scalpel and two dental diode lasers and compare the healing patterns through histological and immunohistochemical analyses. The null hypothesis was that there is no difference in the healing patterns of surgical wounds treated using the steel scalpel and two dental diode lasers. Based on the study results, the null hypothesis was accepted (*p* > 0.05; Table 1). We verified that there was no significant difference in the results of histological and immunohistochemical analyses of surgical wounds treated using a steel scalpel and two dental diode lasers.

Laser refers to light amplification by the stimulated emission of electromagnetic waves. The laser oscillator has mirrors on both sides of the long and thin resonator. When energy is supplied to the laser medium from the outside, light is generated from the medium. It is a powerful laser beam [13]. In the dental field, the ruby laser was first used for therapeutic purposes in 1964, and subsequent studies investigated the effects of CO_2_, Nd:YAG, and Er:YAG lasers [14].

The characteristics of a laser vary depending on the medium that is used as the energy source, and various other characteristics can be expressed by mixing different media [15]. Among them, the dental diode laser uses a solid-state semiconductor as a medium and is generally composed of gallium, arsenic, and a combination of other elements such as aluminum and indium. The wavelength of a surgical dental diode laser that can be used in dentistry ranges from 800 to 980 nm, which is at the beginning of the near-infrared nonvisible, nonionization spectrum [13]. A wavelength of 980 nm was used in this study. As the absorption of light at this wavelength is higher in tissues containing hemoglobin or pigment than in water, it is more suitable for soft tissue surgery than hard tissue treatment [16]. The thermal effect of the dental diode laser is caused by the concentration of heat at the end of the fiber tip. This leads to the formation of a thicker coagulation layer in the treatment area [17].

In the field of periodontology, diode laser is mainly used in two clinical methods other than surgery. The first is to additionally use a diode laser for scaling/root planning. However, it has been reported that there is no evidence of additional microbiological, immunological, and clinical advantages of the additional use of this diode laser compared to SRP alone, which was supported by a meta-analysis study [18,19]. The second method, low-level laser therapy (LLLT), is an alternative treatment method that reduces the inflammatory response of the periodontal tissue and prevents the progression of destructive periodontal disease. In a recent study, it was reported that LLLT using diode laser with a wavelength of 810nm reduced the synthesis of anti-inflammatory cytokines for *P. gingivalis* LPS in human gingival fibroblast [20].

In this study, the changes in the healing procedure were compared 3, 7, and 14 days after epithelial-removed wounds were formed on the rat oral mucosa using a scalpel and two dental diode lasers. Histological analysis of the gingival epithelium and connective tissue was performed, and the presence of inflammatory cells was assessed. Immunohistochemical investigations were performed using iNOS and CD68 as markers. NOS is an enzyme that produces nitric oxide and plays a role in non-specific immune responses. Three NOS isoenzymes have been identified to date, including eNOS, iNOS, and nNOS [12]. CD68 is a cell-penetrating glycoprotein expressed at high levels in monocytic cells (e.g., monocytic phagocytes and osteoclasts) and macrophages, and was used in this study to evaluate the presence of histocytes along with histological examination [7,8,9].

We observed a delayed healing pattern in laser-induced wounds compared to that in surgically induced wounds at all periods. The expression of iNOS did not differ between the three groups on days 3 and 7, but the groups treated using the two dental diode lasers maintained higher expression levels than the group exposed to a steel scalpel on day 14. D’ Arcangelo et al. [1] and Ghalayani et al. [21] performed a study comparing the expression of NOS (eNOS and iNOS) with the healing pattern after forming an incisional wound in the oral cavity of a rat using a dental diode laser and a steel scalpel. D’ Arcangelo et al. showed that both iNOS and eNOS were highly expressed in the laser-irradiated regions. Ghalayani et al. found no significant difference in the level of eNOS, but detected significantly higher iNOS levels in the laser-irradiated area than in the scalpel-exposed area. The association between the slow healing of laser-induced wounds observed in this study and high levels of iNOS is not clear. Nergiz et al. and Fahrad et al. reported that lower iNOS levels were associated with enhanced healing, whereas Schwentker et al. suggested an association between decreased healing and lower iNOS [22,23,24].

The number of macrophages represented by CD68 was similar in the three groups on days 3 and 7; however, after 14 days, their number was higher at the laser-treated sites. During the wound healing process, neutrophils first appear at the wound site and perform phagocytosis. This step is followed by the recruitment of macrophages to purify the wound and promote healing through phagocytosis and cytokine secretion [25]. Although this process occurs in the early stage of wound healing, tissue observation before 3 days was not performed in this study. Therefore, the observation time in this study may be inappropriate for studying the wound-healing process in the inflammatory phase. Further research with varied period settings should be conducted.

In the present study, we visually confirmed that wound healing after the laser procedure was slower than that after the steel scalpel procedure. According to a report based on CO_2_ laser irradiation, oral mucosa wounds made by laser have less collagen formation, less wound contraction, and slower epithelial regeneration than those made by conventional surgical scalpels [26]. The delayed re-epithelialization of wounds by laser can be explained by inhibitory substances produced by the necrotic tissue, physical obstacles due to the presence of eschar, and heat fixation of adjacent epithelial cells [27].

Many advantages of dental diode lasers have been reported in several studies comparing conventional surgery and dental diode lasers. D’ Arcangelo et al. [1] and Amaral et al. [28] concluded that the dental diode laser has many advantages over conventional surgical methods, including less bleeding, less swelling, more favorable clotting, less scarring, no sutures required, shorter surgery time, and less postoperative pain. Bakhtiari et al. [29] reported that dental diode lasers cause less mechanical trauma and immediately disinfect surgical sites. However, Jin et al. also reported that the dental diode laser caused greater tissue damage than the conventional surgical procedure and Er Cr: YSGG laser [30].

Unlike other lasers such as CO_2_ lasers, dental diode laser devices do not require large equipment and offer the advantage of being convenient to use with handy size. The two types of dental diode lasers used in this study also had a small device, and there was no discomfort compared with the surgical procedure of holding and operating the handpiece. The existing commercially available laser device (Laser B) is wireless. However, there is a button to control the output and mode on the handpiece; therefore, there may be a problem with the volume of the handpiece that can affect the operator’s tactile sensitivity. On the other hand, Laser A has the advantage of being lightweight and easy to operate because there is no operation button on the handpiece. In this study, no significant difference in healing was observed using the two dental diode laser devices, potentially because both types of lasers are dental diode lasers and were irradiated with the same mode and similar output. The ease of operation of the laser device may be an important factor when selecting one of several similar laser devices.

In clinical cases, de-epithelization for treatment includes the removal of gingival hyperpigmentation and hypertrophic gingiva. Gingival hyperpigmentation is the deposition of excessive melanin pigment by the action of tyrosinase in melanocytes in the basal layer of the epithelium [31]. Conventional surgical procedures using steel scalpels are considered the gold standard treatment. However, the laser method is commonly used. The wavelength of the diode laser used in the dental field is approximately 800–1000 nm, and the absorption of light at this wavelength is higher in tissues containing hemoglobin or pigment than in water [32]. Recently, diode lasers with various wavelengths have been developed for various dental treatments. Dental diode lasers are expected to be applied in more diverse fields of dentistry in the future.

## 5. Conclusions

Laser-induced wounds tended to heal more slowly than surgical wounds using a steel scalpel, but histological and immunohistochemical results showed no significant difference between the dental diode laser and scalpel groups. In addition, similar histological and immunohistochemical results were observed in the two types of dental diode lasers. Although the power and surgical techniques need to be established through future research, the new laser is expected to be actively used in dental procedures because it has the advantage of manipulation.

## Figures and Tables

**Table 1:** Result of histologic and immunohistochemical analyses.

Day	Analysis	Scalpel Group			Laser A Group			Laser B Group			p *
3	Neutrophils	1	0	0	3	1	0	0	0	3	0.656
	Lymphocytes	0	1	1	1	1	0	0	0	0	0.202
	Histocytes (CD68)	2	2	3	3	1	3	1	3	3	0.960
	iNOS-positive cells	3	2	2	3	3	3	3	3	3	0.102
7	Neutrophils	0	0	0	0	0	1	0	0	0	0.368
	Lymphocytes	1	1	0	1	0	1	1	1	1	0.565
	Histocytes (CD68)	2	1	2	2	2	2	2	1	3	0.740
	iNOS-positive cells	2	2	2	2	2	3	1	2	2	0.264
14	Neutrophils	0	0	0	0	0	2	0	0	0	0.368
	Lymphocytes	1	1	1	1	1	1	1	0	0	0.102
	Histocytes (CD68)	1	2	1	2	3	3	2	3	3	0.087
	iNOS-positive cells	1	2	1	2	3	3	2	3	3	0.087
p **		0.457			0.488			0.811			

Scores in this field at 100× magnification were classified according to the following criteria: 0 (0 cells/HPF), 1 (1–30 cells/HPF), 2 (31–60 cells/HPF), and 3 (more than 61 cells/HPF). Significance was determined by the Kruskal–Wallis H test (*, comparison among scalpel group, laser A group, and laser B group; **, comparison among 3, 7, and 14 days), *p* < 0.05.

## Data Availability

The datasets used and/or analyzed during the current study are available from the corresponding author upon reasonable request.
